# Supplementary material for: Structural basis for genome packaging, retention, and ejection in human cytomegalovirus
Source: Nat Commun. 2021 Jul 27;12:4538. doi: 10.1038/s41467-021-24820-3 (PMC8316551; doi:10.1038/s41467-021-24820-3)
Supplement: Supplementary file 1 — Supplementary information [file 41467_2021_24820_MOESM1_ESM.pdf]

Supplementary Information

Structural Basis for Genome Packaging, Retention and Ejection in Human Cytomegalovirus

Zhihai Li, Jingjing Pang, Lili Dong and Xuekui Yu

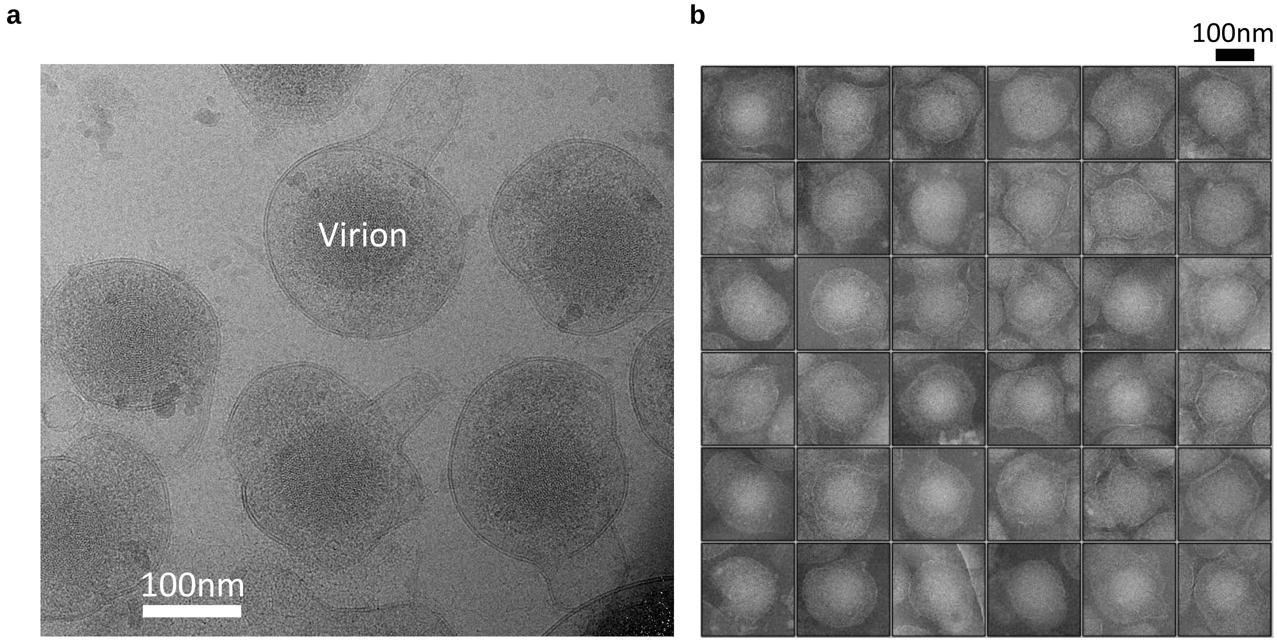

Supplementary Fig. 1 CryoEM micrograph and particle images of HCMV virion. a Representative micrograph recorded with a 300 kV Titan Krios microscope. The particle distribution and morphology are similar among all the selected micrographs used for the final structure determination. **b** Representative particle images selected from those used for the final reconstruction of the HCMV capsid, showing the intact envelope of the virion.

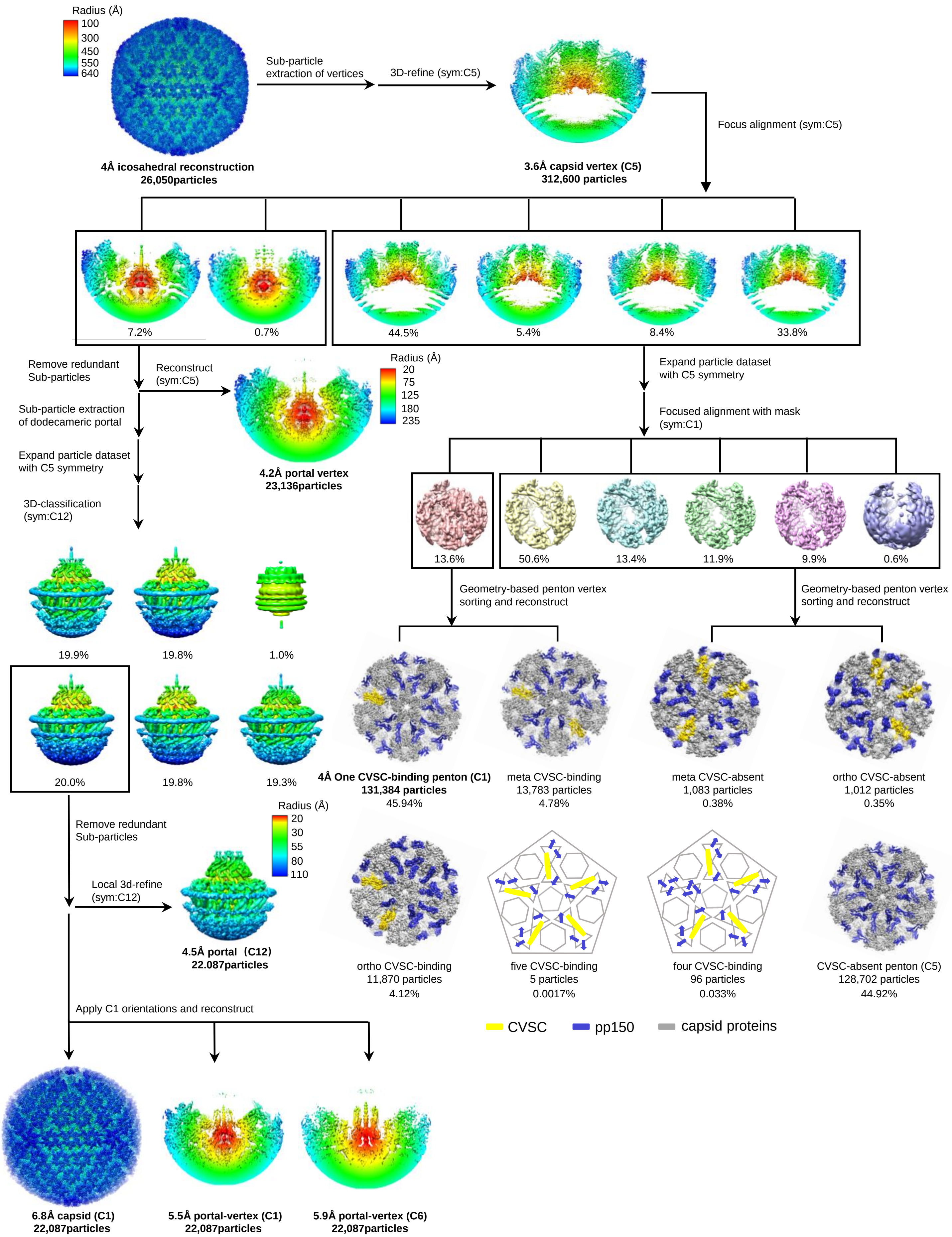

Radius (Å)
100
300
450
550
640
4Å icosahedral reconstruction
26,050particles
Sub-particle
extraction of vertices
3D-refine (sym:C5)
3.6Å capsid vertex (C5)
312,600 particles
Focus alignment (sym:C5)
7.2%
0.7%
44.5%
5.4%
8.4%
33.8%
Remove redundant
Sub-particles
Reconstruct
(sym:C5)
Radius (Å)
20
75
125
180
235
4.2Å portal vertex
23,136particles
Sub-particle extraction
of dodecameric portal
Expand particle dataset
with C5 symmetry
3D-classification
(sym:C12)
19.9%
19.8%
1.0%
20.0%
19.8%
19.3%
Expand particle dataset
with C5 symmetry
Focused alignment with mask
(sym:C1)
13.6%
50.6%
13.4%
11.9%
9.9%
0.6%
Geometry-based penton vertex
sorting and reconstruct
Geometry-based penton vertex
sorting and reconstruct
4Å One CVSC-binding penton (C1)
131,384 particles
45.94%
meta CVSC-binding
13,783 particles
4.78%
meta CVSC-absent
1,083 particles
0.38%
ortho CVSC-absent
1,012 particles
0.35%
Remove redundant
Sub-particles
Radius (Å)
20
30
55
80
110
Local 3d-refine
(sym:C12)
4.5Å portal（C12）
22.087particles
ortho CVSC-binding
11,870 particles
4.12%
five CVSC-binding
5 particles
0.0017%
four CVSC-binding
96 particles
0.033%
CVSC-absent penton (C5)
128,702 particles
44.92%
CVSC
pp150
capsid proteins
Apply C1 orientations and reconstruct
6.8Å capsid (C1)
22,087particles
5.5Å portal-vertex (C1)
22,087particles
5.9Å portal-vertex (C6)
22,087particles

Supplementary Fig. 2 Image processing workflow for the HCMV sub-particles and asymmetric capsid from the intact virion. The reconstructions of capsid, the clipped vertices and dodecameric portal are radially colored. For the classified CVSC-binding penton vertices, the reconstructions are colored by CVSC (yellow), pp150 (blue) and capsid proteins (gray). Because the cryoEM structures of the four and the five CVSC-binding penton vertices can not be reconstructed with only a few particles, we used two diagrams to illustrate these two penton vertices.

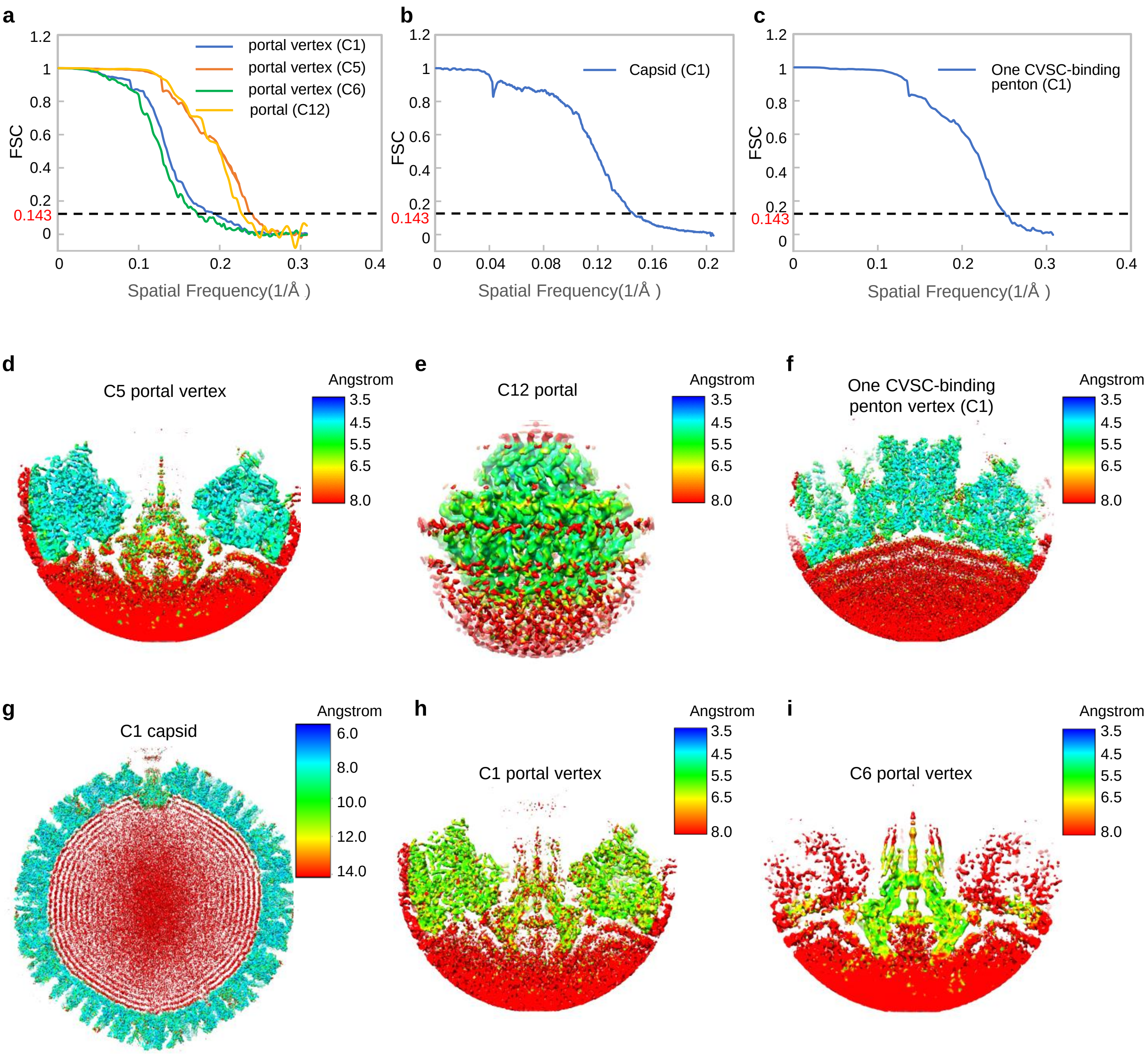

Supplementary Fig. 3 Global and local resolution assessments of reconstructions of the sub-particles and asymmetric capsid from the virion capsid. a-c Gold-standard FSC curves of cryoEM reconstructions of C1 portal vertex (5.5 Å), C6 portal vertex (5.9 Å), the C5 portal vertex (4.2 Å), the C12 portal main body (4.5 Å), C1 capsid (6.8 Å) and C1 one CVSC-binding penton vertex (4.0 Å). **d-i** Local resolution distributions of density maps estimated by ResMap.

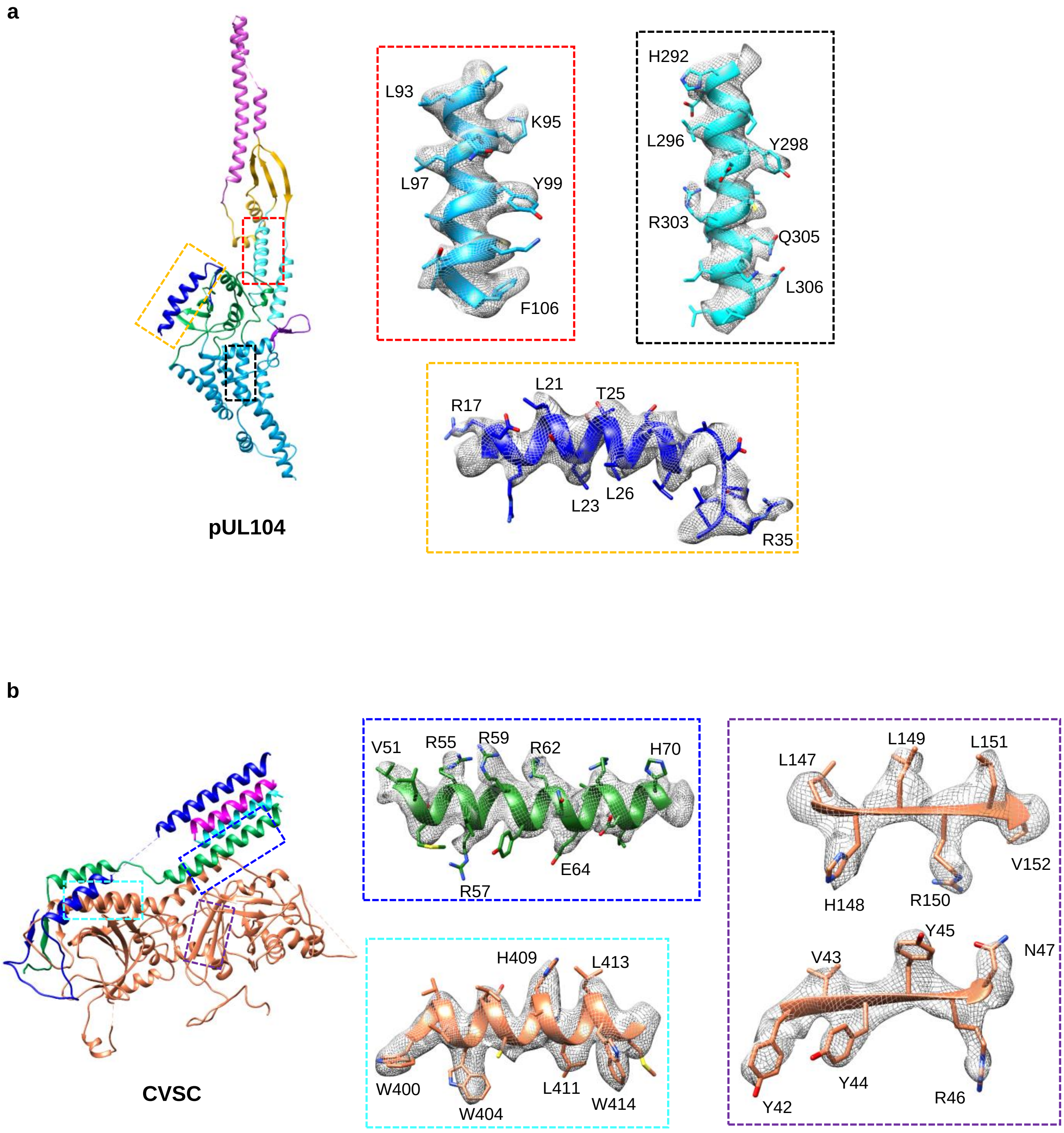

Supplementary Fig. 4 Density maps and atomic models of pUL104 (a) and CVSC (b). Insets are the zoomed-in views of the boxed regions, showing the residue side chains in the density maps.

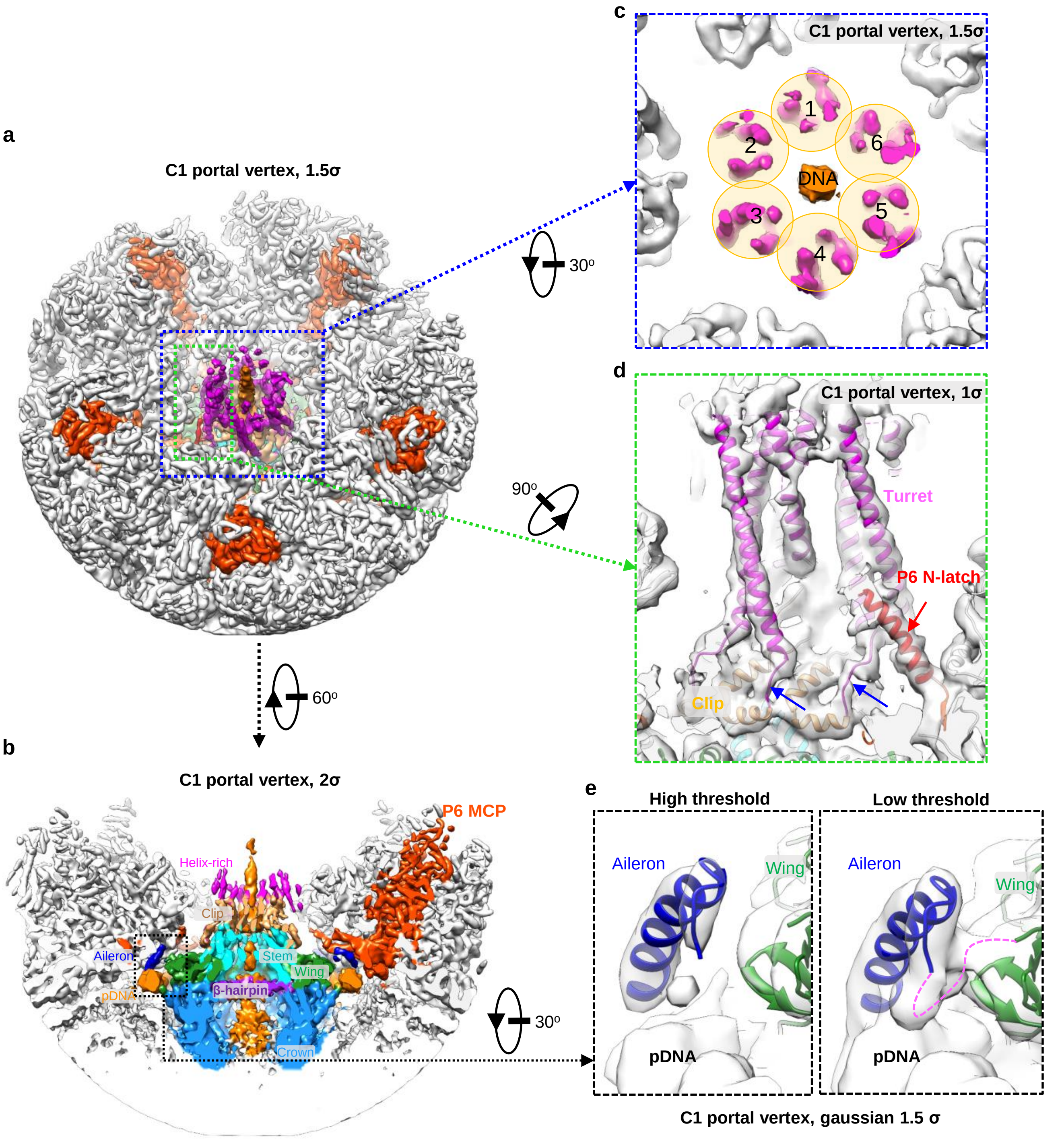

Supplementary Fig. 5 Reconstruction of the C1 portal vertex. a-b Overview (a) and clipped view (b) of the C1 density map of the portal vertex. The portal is colored by domain, as indicated. The pDNA and the P6 MCP are in orange and orange-red, respectively. The P6 N-latch is highlighted in red. **c** Top view of the blue boxed region in **a**, showing that the portal turret has a 6-fold symmetry. **d** Density map and atomic model of the green boxed region in **a**, showing the connection between the portal turret and the clip, as indicated by the blue arrows. **e** Density map and atomic model of the boxed region in **b**, showing the connecting density between one aileron domain and the wing domain of the portal when displayed at a low threshold.

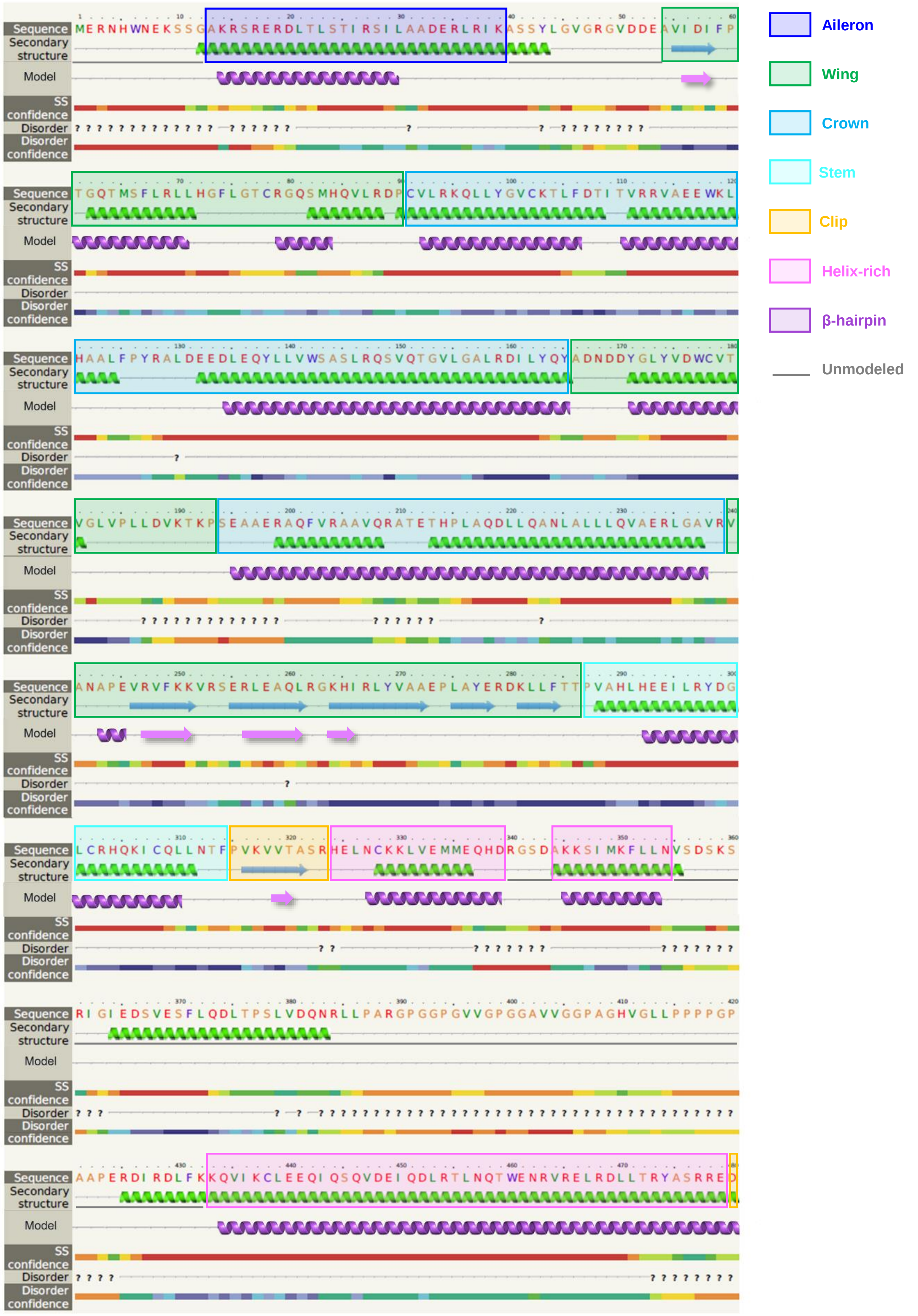
Sequence
Secondary structure
Model
SS confidence
Disorder
Disorder confidence
Aileron
Wing
Crown
Stem
Clip
Helix-rich
β-hairpin
Unmodeled

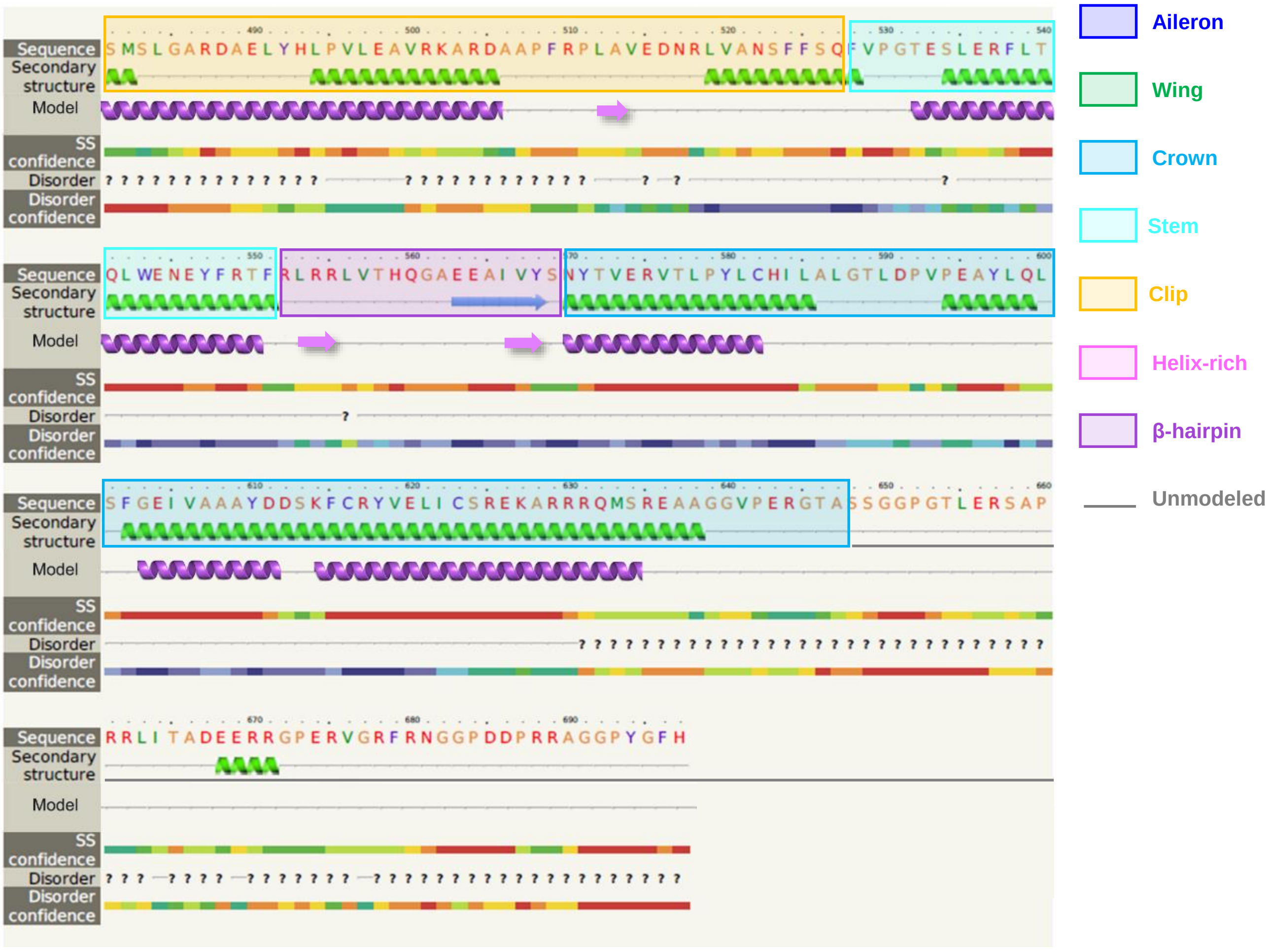

Supplementary Fig. 6 Secondary structure predication of the pUL104 protein.

The secondary structure prediction of pUL104 was performed using *Phyre2*. The model of pUL104 resolved in this study is in purple.

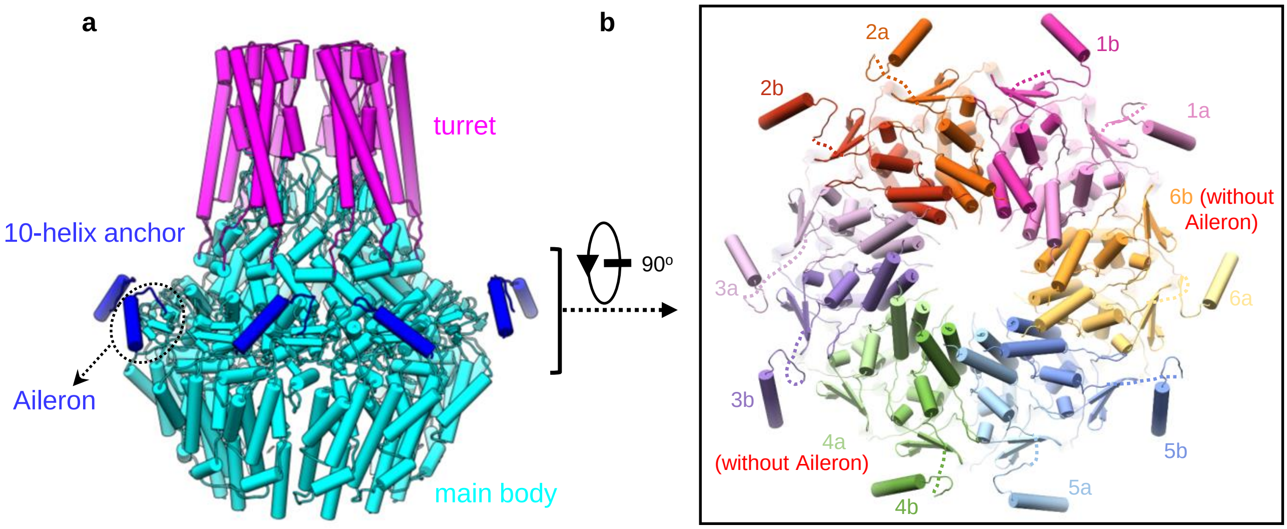

Supplementary Fig. 7 The structure of the portal. a Side view of the portal. The C6 turret, the C12 main body, and the C5 10-helix anchor are in magenta, cyan, and blue, respectively. **b** Top view of the portal (colored by molecule), showing the tentative identity assignments of the 10 aileron domains with respect to the 12 portal main bodies.

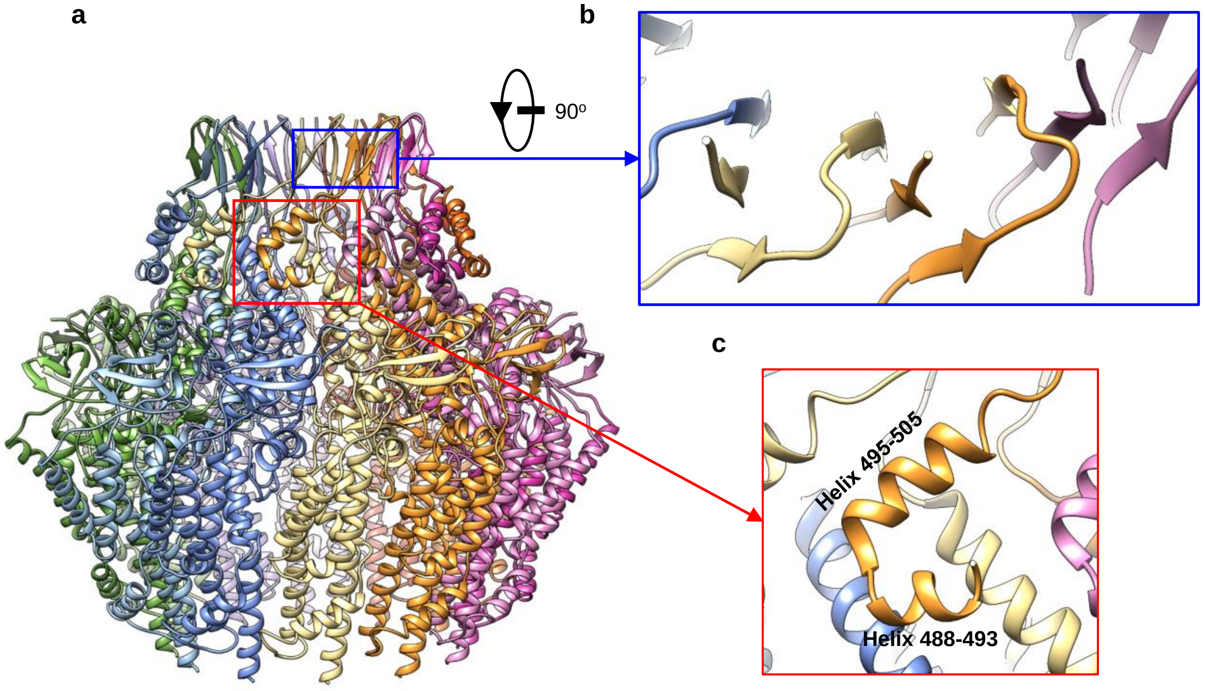

Supplementary Fig. 8 The structure of the portal main body and the inter-monomer interactions. a Atomic model of the portal main body, colored by molecule. **b-c** Zoomed-in views of the boxed regions in **a**, showing the β augmentations in the clip region (b) and the two clip helices from each monomer holding the two neighboring ones (c).

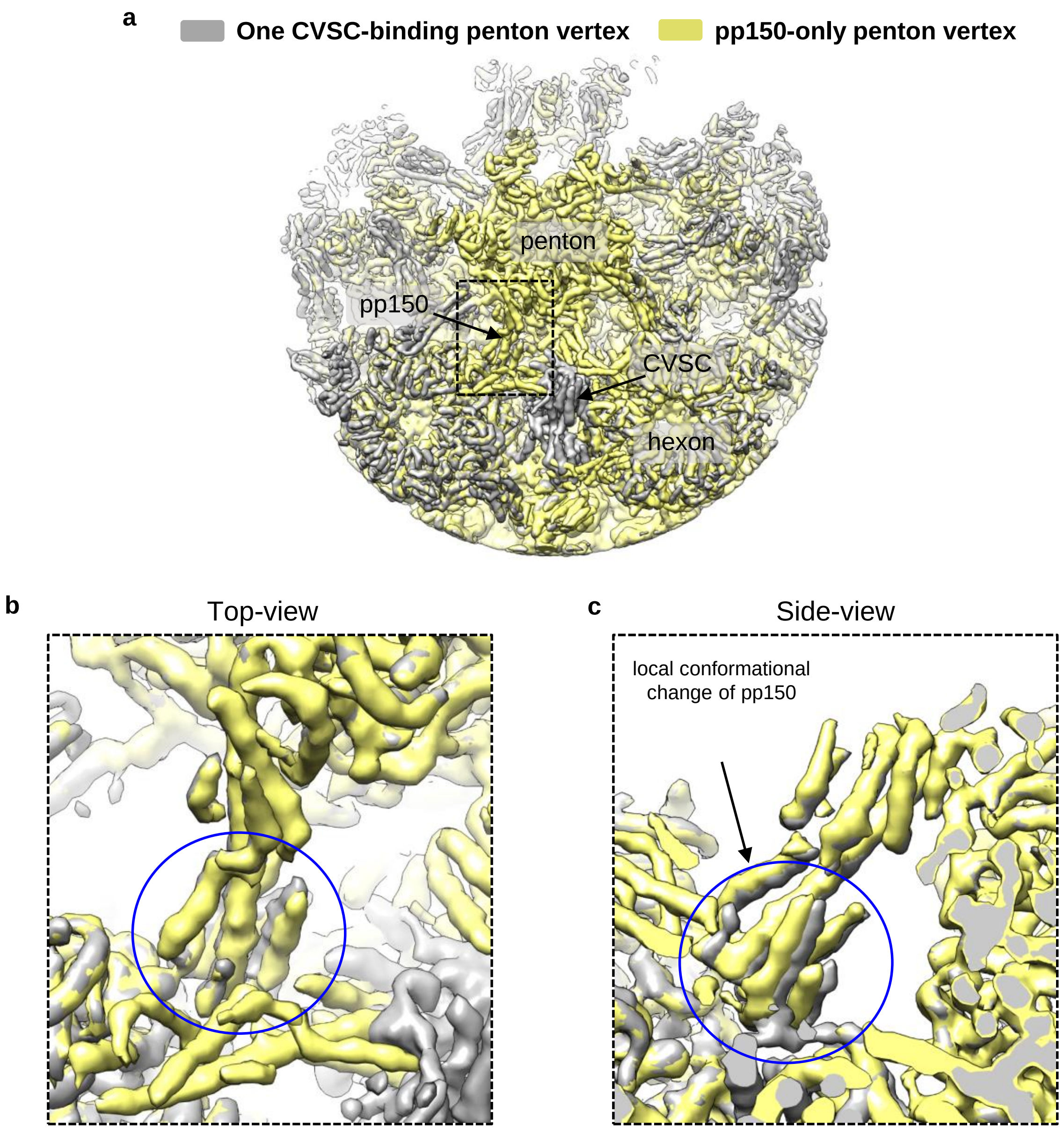

Supplementary Fig. 9 Structural difference of the two corresponding pp150s from the pp150-only register and CVSC-binding register. a Superimposition of one CVSC-binding vertex (gray) and the pp150-only (yellow) penton vertex. **b-c** Top (b) and side (c) views of the boxed region in **a**, showing the structural changes at the lower region of the pp150 (yellow) sitting atop the rotated Ta as compared with that (gray) atop the unrotated one.

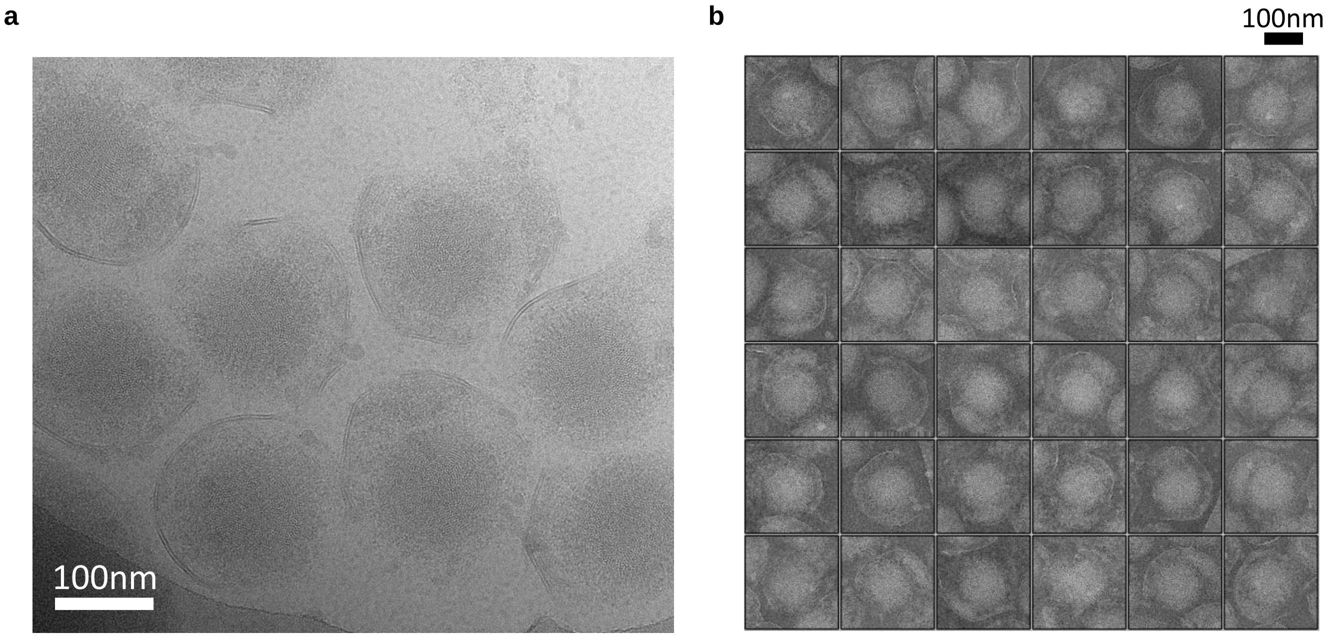

Supplementary Fig. 10 CryoEM micrograph and particle images of the partially-enveloped nucleocapsid. **a** Representative micrograph of the partially-enveloped nucleocapsid. The particle distribution and morphology are similar among all the selected micrographs used for the final structure determination. **b** Representative particle images selected from those used for the final reconstruction, showing the partially solubilized envelopes of the viral particles.

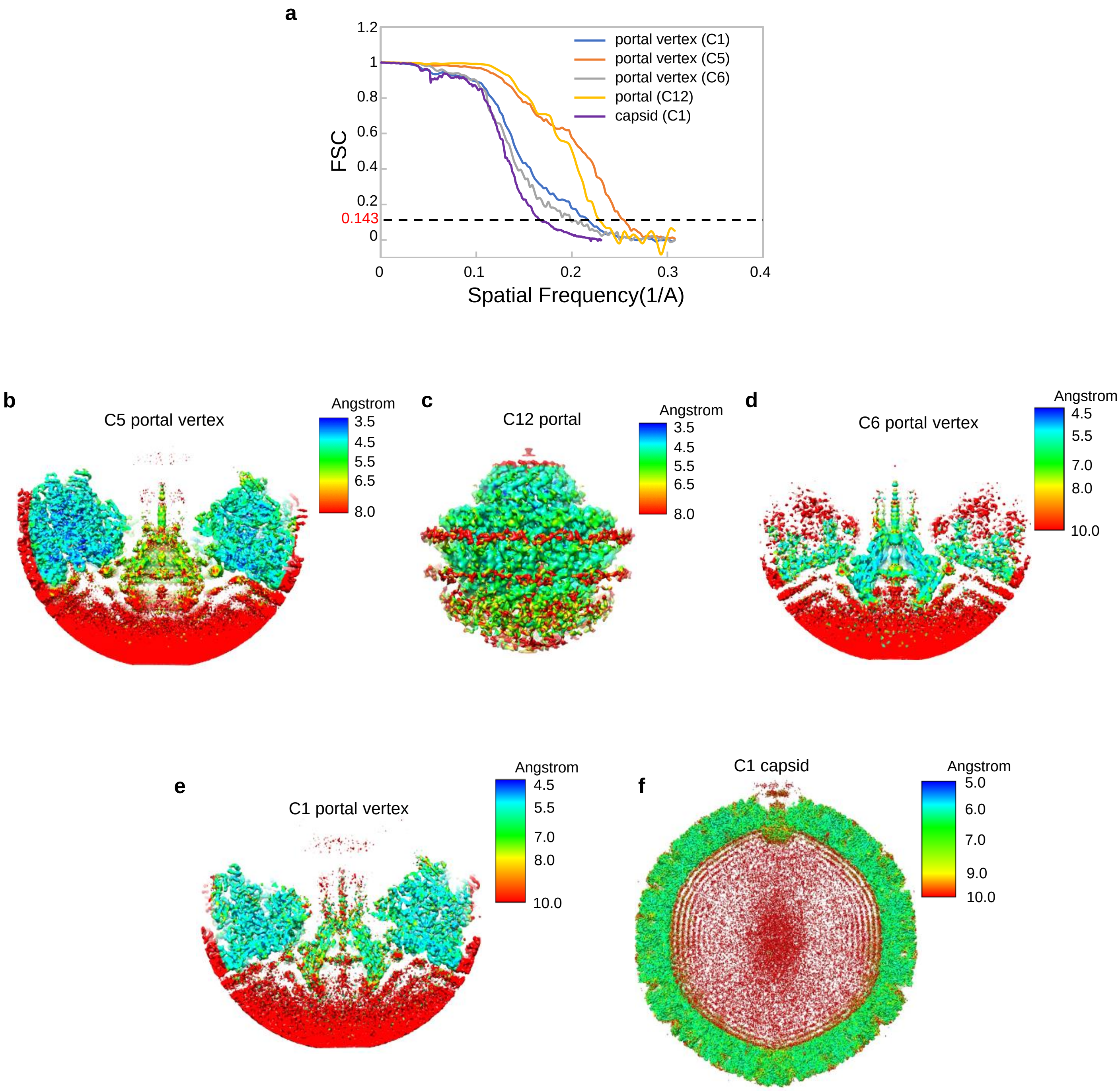

Supplementary Fig. 11 Global and local resolution assessments of the sub-particles and the asymmetric capsid reconstructions from the partially-enveloped nucleocapsid. a Gold-standard FSC curves of the reconstructions of the C5 portal vertex (4.0 Å), the C12 portal main body (4.2 Å), the C1 portal vertex (4.8 Å), the C6 portal vertex (5.3 Å), and the C1 capsid (6.4 Å). **b-f** Local resolution distributions of the density maps estimated by ResMap.

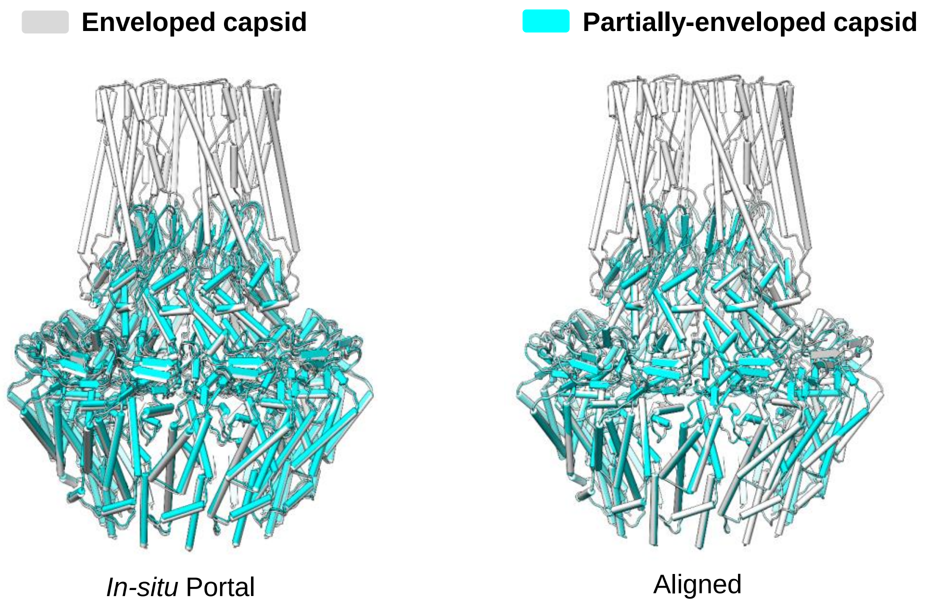

Supplementary Fig. 12 Conformational changes of the portal caused by rupture of the virion envelope. Superimpositions of the portals from the intact virion and the partially-enveloped nucleocapsid without (left) and with alignment (right).

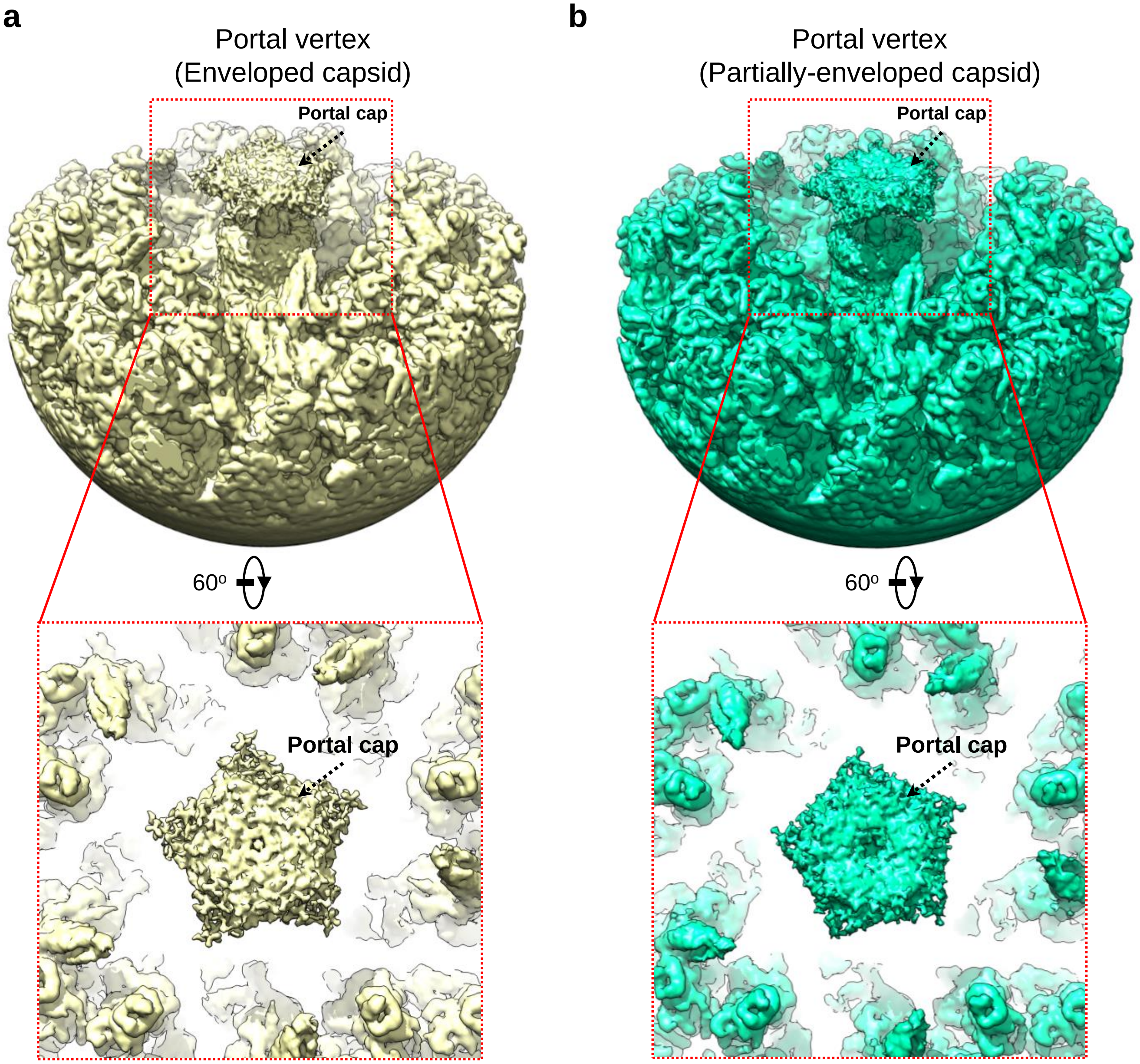

Supplementary Fig. 13 Structural comparison of the portal vertex between enveloped (a) and partially-enveloped capsids (b). *Insets*, Top views of the boxed region of the portal vertex, showing the portal cap essentially unchanged after rupture of the virion envelope.

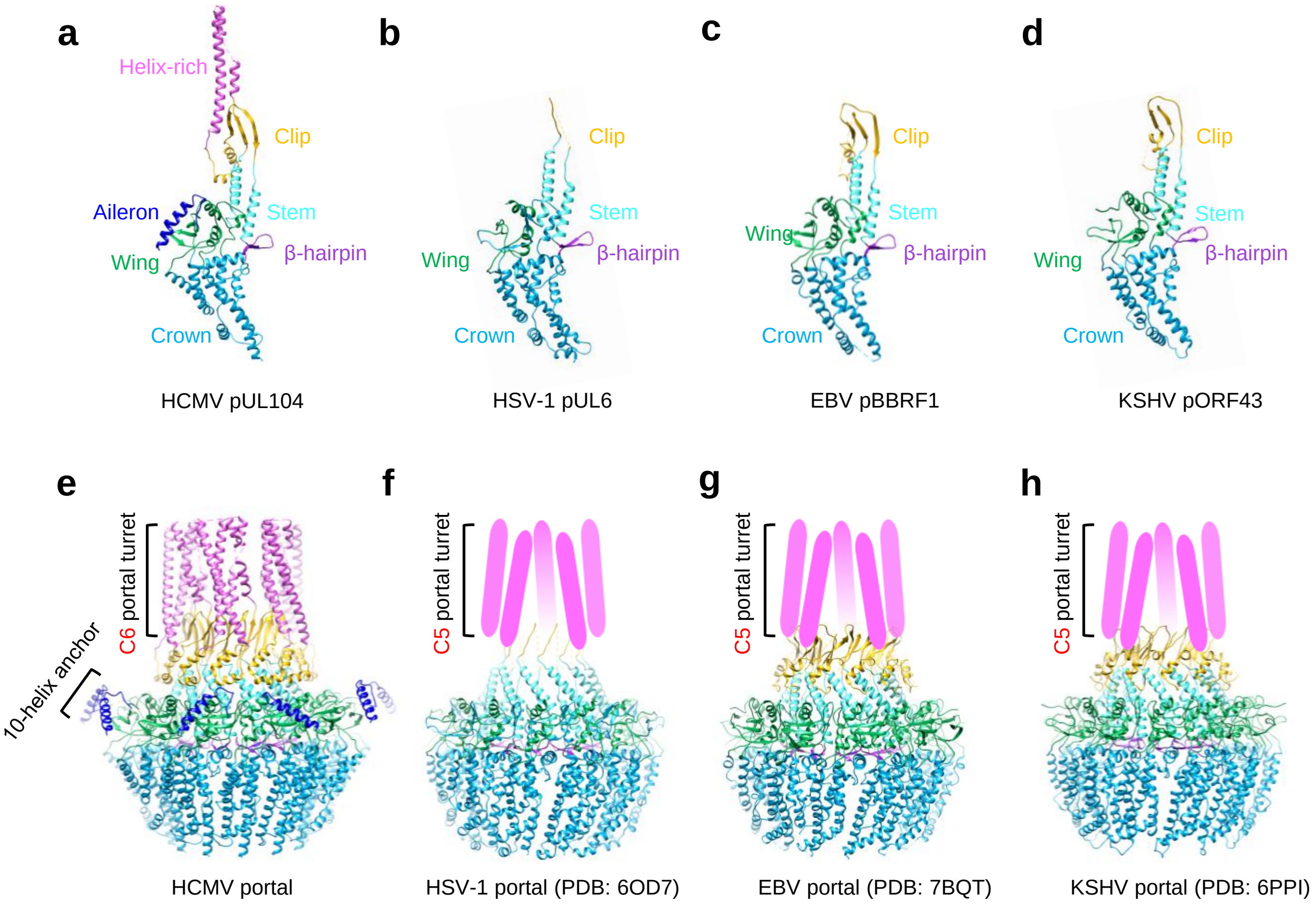

Supplementary Fig. 14 Structural comparison of the portals from the different herpesviruses, including HCMV, HSV-1, EBV, and KSHV. a-d Atomic models of the portal proteins from HCMV (a), HSV-1 (b), EBV (c) and KSHV (d). **e-h** Structures of the in situ portals from HCMV (e), HSV-1 (f), EBV (g) and KSHV (h). The portal turret of HCMV is arranged with 6-fold symmetry, whereas the counterparts of the other three herpesviruses are uniformly assembled with 5-fold symmetry.

Supplementary Table. 1 Cryo-EM data collection, image processing, and refinement statistics

	Virion capsid						Partially-enveloped capsid				
	C5 portal vertex (EMD: 31297; PDB: 7ET3)	C6 portal vertex (EMD: 31299; PDB: 7ETM)	C12 portal (EMD: 31295)	C1 portal vertex (EMD: 31290)	C1 capsid (EMD: 31292)	C1 CVSC-binding penton vertex (EMD: 31301; PDB: 7ETO)	C5 portal vertex (EMD: 31298; PDB: 7ETJ)	C6 portal vertex (EMD: 31300)	C12 portal (EMD: 31296; PDB: 7ET2)	C1 portal vertex (EMD: 31291)	C1 capsid (EMD: 31293)
**Data collection and processing**											
Voltage (kV)	300	300	300	300	300	300	300	300	300	300	300
Electron exposure (e–/Å^2)	30	30	30	30	30	30	30	30	30	30	30
Defocus range (μm)	-0.5 to -2.0	-0.5 to -2.0	-0.5 to -2.0	-0.5 to -2.0	-0.5 to -2.0	-0.5 to -2.0	-1.0 to -2.5	-1.0 to -2.5	-1.0 to -2.5	-1.0 to -2.5	-1.0 to -2.5
Pixel size (Å)	1.625	1.625	1.625	1.625	1.625	1.625	1.625	1.625	1.625	1.625	1.625
Symmetry imposed	C5	C6	C12	C1	C1	C1	C5	C6	C12	C1	C1
Initial particle images (no.)	24,695	23,136	23,136	23,136	26,050	285,935	48,194	42,849	42,849	42,849	49,963
Final particle images (no.)	23,136	22,087	22,087	22,087	22,087	131,384	42,849	40,903	40903	40,903	40,903
Map resolution (Å)	4.2	5.9	4.5	5.5	6.8	4.0	4.0	5.3	4.2	4.8	6.3
FSC threshold	0.143	0.143	0.143	0.143	0.143	0.143	0.143	0.143	0.143	0.143	0.143
Map resolution range (Å)	3.5-8.0	3.5-8.0	3.5-8.0	3.5-8.0	6.0-14.0	3.5-8.0	3.5-8.0	4.5-10.0	3.5-8.0	4.5-10.0	5.0-10.0
**Refinement**											
Model resolution (Å)	4.3	6.8	n/a	n/a	n/a	4.1	4.1	n/a	4.3	n/a	n/a
FSC threshold	0.5	0.5				0.5	0.5		0.5		
Model resolution range (Å)	4.3-66	6.8-50				4.1-50	4.1-50		4.3-66		
Map sharpening *B* factor (Å^2)	-171	-229	-400	-132	-373	-172	-50	-121	-185	-50	-127
Model composition			n/a	n/a	n/a			n/a		n/a	n/a
Non-hydrogen atoms	86,327	45,246				98,743	86,613		38,880		
Protein residues	10,842	5,970				12,394	10,879		4,812		
Ligands	0	0				0	0		0		
B factors (Å^2)											
Protein	50	276				58	156		59		
Ligand											
R.m.s. deviations											
Bond lengths (Å)	0.004	0.008				0.007	0.005		0.006		
Bond angles (°)	0.876	1.512				1.000	0.923		1.031		
Validation											
MolProbity score	1.76	2.08				1.88	1.79		1.67		
Clashscore	6.61	10.72				8.44	8.09		6.45		
Poor rotamers (%)	0.42	1.86				0.75	0.41		0.41		
Ramachandran plot											
Favored (%)	94.11	95.35				93.64	95.01		95.53		
Allowed (%)	5.89	4.44				6.36	4.99		4.47		
Disallowed (%)	0.00	0.20				0.00	0.00		0.00		
