# Supplementary material for: Structural basis for genome packaging, retention, and ejection in human cytomegalovirus
Source: Nat Commun. 2021 Jul 27;12:4538. doi: 10.1038/s41467-021-24820-3 (PMC8316551; doi:10.1038/s41467-021-24820-3)
Supplement: Supplementary file 3 — Reporting Summary [file 41467_2021_24820_MOESM3_ESM.pdf]

nature portfolio

Corresponding author(s): Xuekui Yu

Last updated by author(s): Jun 22, 2021

Reporting Summary

Nature Portfolio wishes to improve the reproducibility of the work that we publish. This form provides structure for consistency and transparency in reporting. For further information on Nature Portfolio policies, see our Editorial Policies and the Editorial Policy Checklist.

Statistics

For all statistical analyses, confirm that the following items are present in the figure legend, table legend, main text, or Methods section.

n/a	Confirmed	
☒	☐	The exact sample size (n) for each experimental group/condition, given as a discrete number and unit of measurement
☐	☒	A statement on whether measurements were taken from distinct samples or whether the same sample was measured repeatedly
☒	☐	The statistical test(s) used AND whether they are one- or two-sided<br>*Only common tests should be described solely by name; describe more complex techniques in the Methods section.*
☒	☐	A description of all covariates tested
☒	☐	A description of any assumptions or corrections, such as tests of normality and adjustment for multiple comparisons
☒	☐	A full description of the statistical parameters including central tendency (e.g. means) or other basic estimates (e.g. regression coefficient) AND variation (e.g. standard deviation) or associated estimates of uncertainty (e.g. confidence intervals)
☒	☐	For null hypothesis testing, the test statistic (e.g. F, t, r) with confidence intervals, effect sizes, degrees of freedom and P value noted<br>*Give P values as exact values whenever suitable.*
☒	☐	For Bayesian analysis, information on the choice of priors and Markov chain Monte Carlo settings
☒	☐	For hierarchical and complex designs, identification of the appropriate level for tests and full reporting of outcomes
☒	☐	Estimates of effect sizes (e.g. Cohen's d, Pearson's r), indicating how they were calculated

Our web collection on statistics for biologists contains articles on many of the points above.

Software and code

Policy information about availability of computer code

Data collection	SerialEM v3.7
Data analysis	Relion v3.0, MotionCor2 v1.2.2, CTFFIND4, PHENIX-1.14, COOT 0.8.9.1, Chimera v1.14, ResMap v1.1.4, Phyre v2.0

For manuscripts utilizing custom algorithms or software that are central to the research but not yet described in published literature, software must be made available to editors and reviewers. We strongly encourage code deposition in a community repository (e.g. GitHub). See the Nature Portfolio guidelines for submitting code & software for further information.

Data

Policy information about availability of data

All manuscripts must include a data availability statement. This statement should provide the following information, where applicable:

- Accession codes, unique identifiers, or web links for publicly available datasets
- A description of any restrictions on data availability
- For clinical datasets or third party data, please ensure that the statement adheres to our policy

All density maps have been deposited in the Electron Microscopy Bank (https://www.ebi.ac.uk/pdbe/emdb/) under accession codes EMD-31297 (C5 portal vertex from the intact virion), EMD-31295 (C12 portal from the intact virion), EMD-31292 (C1 capsid from the intact virion), EMD-31290 (C1 portal vertex from the intact virion), EMD-31299 (C6 portal vertex from the intact virion), EMD-31301 (C1 one CVSC-binding penton vertex from the intact virion), EMD-31298 (C5 portal vertex from the partially-enveloped nucleocapsid), EMD-31296 (C12 portal from the partially-enveloped nucleocapsid), EMD-31293 (C1 capsid from the partially-enveloped nucleocapsid), EMD-31291 (C1 portal vertex from the partially-enveloped nucleocapsid) and EMD-31300 (C6 portal vertex from the partially-enveloped nucleocapsid). The atomic coordinates have been deposited in the Protein Data Bank (http://www.rcsb.org) under accession code 7ET2 (C12 portal), 7ETM (C6 portal), 7ET3 (C5 portal vertex from the intact virion), 7ETJ (C5 portal vertex from the partially-enveloped nucleocapsid) and 7ETO (C1 one CVSC-binding penton

vertex).

Field-specific reporting

Please select the one below that is the best fit for your research. If you are not sure, read the appropriate sections before making your selection.

- [x] Life sciences
- [ ] Behavioural & social sciences
- [ ] Ecological, evolutionary & environmental sciences

For a reference copy of the document with all sections, see nature.com/documents/nr-reporting-summary-flat.pdf

Life sciences study design

All studies must disclose on these points even when the disclosure is negative.

Sample size	No statistical method was used to determine sample size.
Data exclusions	Micrographs with poorly fitted CTFs were excluded from further image processing.
Replication	No experiments were performed with replication.
Randomization	For 3D refinement in Relion v3.0, the particle dataset was randomly divided into two subsets. Otherwise, no randomization was performed.
Blinding	Experimental results did not require any subjective analysis, thus no experiments were preformed with blinding

Reporting for specific materials, systems and methods

We require information from authors about some types of materials, experimental systems and methods used in many studies. Here, indicate whether each material, system or method listed is relevant to your study. If you are not sure if a list item applies to your research, read the appropriate section before selecting a response.

Materials & experimental systems

n/a	Involved in the study	
x		Antibodies
	x	Eukaryotic cell lines
x		Palaeontology and archaeology
x		Animals and other organisms
x		Human research participants
x		Clinical data
x		Dual use research of concern

Methods

n/a	Involved in the study	
x		ChIP-seq
x		Flow cytometry
x		MRI-based neuroimaging

Eukaryotic cell lines

Policy information about cell lines

Cell line source(s)	Human fibroblast MRC-5 (ATCC No. CCL-171)
Authentication	No further authentication was performed for commercially available cell lines.
Mycoplasma contamination	No mycoplasma contamination.
Commonly misidentified lines (See ICLAC register)	No commonly misidentified cell lines were used.
